# Referrals for pediatric weight management: the importance of proximity

**DOI:** 10.1186/1472-6963-10-302

**Published:** 2010-11-01

**Authors:** Kathryn A Ambler, Douglas WJ Hagedorn, Geoff DC Ball

**Affiliations:** 1 Department of Pediatrics, Faculty of Medicine & Dentistry, University of Alberta, Edmonton, AB, Canada; 2 Pediatric Centre for Weight and Health, Alberta Health Services, Edmonton, AB, Canada; 3 Department of Geography, Faculty of Arts, University of Calgary, Calgary, AB, Canada

## Abstract

**Background:**

Limited access to weight management care can have a negative impact on the health and well-being of obese children and youth. Our objectives were to describe the characteristics of clients referred to a pediatric weight management centre and explore potential differences according to proximity.

**Methods:**

All demographic and anthropometric data were abstracted from standardized, one-page referral forms, which were received by a pediatric weight management centre in Edmonton, AB (Canada) between April, 2005 and April, 2009.

**Results:**

Referrals (n = 555; 52% male; age [mean +/- standard deviation]: 12.4 +/- 2.6 y; BMI: 32.3 +/- 6.8 kg/m2; BMI percentile: 98.4 +/- 1.7; BMI z-score: 2.3 +/- 0.4) were received from 311 physicians. Approximately 95% of referrals were for boys and girls classified as *obese *or *very obese*. Based on postal code data, individuals were dichotomized as either living within (local; n = 455) or beyond (distant; n = 100) the Edmonton Census Metropolitan Area. Numerous families resided several hundred kilometres away from our centre. Overall, distant clients were taller, weighed more, and were more overweight than their local counterparts. For distant clients, the degree of overweight was higher in youth *versus *children.

**Conclusion:**

Pediatric weight management services must be designed to optimize access to health services, especially for distant clients who may be at increased obesity-related health risk.

## Background

Children living in rural settings are at increased risk of obesity compared to their urban peers [1,2]. This dichotomy is relevant for clients seeking weight management care since specialty clinics tend to be based in metropolitan areas. When rural clients require specialized care for complex conditions such as obesity, living in a remote location can pose challenges [3]. Clinicians practicing in rural communities usually have limited contact with subspecialist colleagues and fewer opportunities for continuing professional education to enhance knowledge and skills [4]. Rural families often have fewer local resources to help them manage health risks that tend to accompany obesity [5]. Despite known inequities in health care access across urban and rural areas, there has been little investigation of how best to align health services delivery for clients based on their geographic proximity to health care centres. The health consequences of pediatric obesity [6] make it critical to better understand how health services can be optimized for families, regardless of whether they live close to or far from specialized pediatric weight management centres. As an initial step to address this knowledge gap, the objectives of this study were to describe the characteristics of clients referred to a pediatric weight management centre and explore potential differences according to proximity. We hypothesized that the severity of obesity would be greater for clients who lived remotely *versus *locally.

## Methods

This research was approved by the Health Research Ethics Board at the University of Alberta and conducted through the Pediatric Centre for Weight and Health (PCWH), a weight management centre affiliated with the Stollery Children's Hospital (Edmonton, AB). The geographic area (>550,000 km^2^) served by this hospital is one of the largest in Canada; in total, >400,000 children and youth reside in this vast region. Referrals are accepted from physicians for 7.5-18.0 year olds with a body mass index (BMI) ≥85^th ^percentile [7].

Birth and referral dates, sex, height, weight, and location of residence data were abstracted from standardized referral forms received from April, 2005 to April, 2009. Although many physicians reported BMI and BMI percentile data, we recalculated these indices using *EpiInfo v3.3 *(Centers for Disease Control and Prevention; Atlanta, GA). Clients were subsequently grouped into the following BMI percentile categories: (a) ≥85^th ^to <95^th ^(*overweight*), (b) ≥95^th ^to <99^th ^(*obese*), or (c) ≥99^th ^(*very obese*).

Using forward sortation area data (postal code records) from each client's reported residence and a national dataset [8], clients were categorized by location as either local (within Edmonton Census Metropolitan Area; ECMA) or distant (beyond ECMA). ECMA was defined as the City of Edmonton plus local counties (Sturgeon, Strathcona, Leduc, and Parkland; total population ≈ 1,000,000). The spatial distribution of referred clients was graphically depicted using *ArcMap 9.3.1 *(ESRI Inc., Redlands, CA) by placing a marker for each recorded postal code at its Single Link Indicator [SLI] coordinates. SLI coordinates establish a unique geographic location that targets the highest concentration of dwellings within a broad region containing all the addresses linked to a particular postal code. While the size of the region that each SLI coordinate corresponds to may vary between urban and rural areas depending on dwelling proximity, this reference coordinate provides a suitably accurate location for analysis without defining discrete dwelling locations; this approach helps to maintain anonymity while retaining meaningful information for graphical presentation. For our statistical procedures, we used multivariate analysis of variance to examine the main effects of sex (boys *vs *girls), age group (children [7.5-12 years old] *vs *youth [13-18 years old), proximity (within ECMA *vs *beyond ECMA), and their interactions on demographic and anthropometric variables. Analyses were performed using *SPSS v17.0 *(SPSS Inc.; Chicago, IL).

## Results

From 2005-2009, 311 physicians submitted 680 referrals to the PCWH. After some referrals were excluded (e.g., incomplete anthropometry data [n = 62], age <7.5 or >18.0 years [n = 22], duplicate referrals [n = 20], BMI <85^th ^percentile [n = 11], and missing postal code data [n = 10]), our dataset included 555 clients. A relatively even distribution of referrals was received for boys (n = 286; 52%) and girls (n = 269; 48%) with boys having a higher BMI percentile and BMI z-score compared to girls (both p < 0.01). Comparing children (n = 333; 60%) to youth (n = 222; 40%) revealed significant differences in height, weight, and BMI (all p < 0.001), but not BMI percentile or BMI z-score (both p > 0.05). The distribution of clients according to BMI percentile categories was as follows: (a) ≥85^th ^to <95^th ^percentile: n = 30 (5.4%); (b) ≥95^th ^to <99^th ^percentile: n = 260 (46.8%); (c) ≥99^th ^percentile: n = 265 (47.7%).

**Figure 1:**
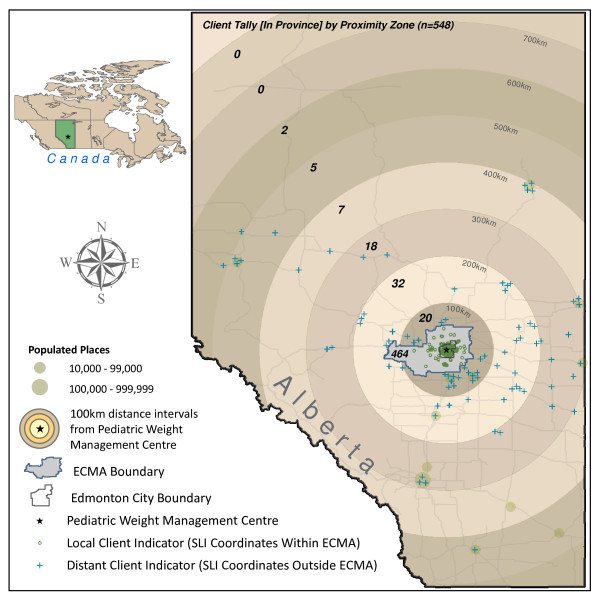
**Spatial distribution of clients referred to the pediatric weight management centre from April, 2005 to April, 2009 from within and beyond the Edmonton Census Metropolitan Area (ECMA) presented using Single Link Indicator (SLI) coordinates**. Note: Of the 555 referrals received by the pediatric weight management centre, 548 are graphically depicted in Figure 1. An additional seven referrals were received for clients living outside the province of Alberta.

Most clients lived within ECMA (n = 455; 82.0%) (Figure 1). Although EMCA includes neighbourhoods that vary by land use and socioeconomic status, there was no discernable geographic pattern to the referrals. Of those living beyond ECMA (n = 100; 18.0%), 72 clients lived more than a one hour (by automobile) from the clinic and seven lived outside the province. Clients who resided beyond ECMA were taller, weighed more, and were more overweight than their peers living within ECMA (Table 1). Analyses also revealed a significant proximity × age group interaction; although no age group differences emerged within ECMA, for those clients living beyond ECMA, youth had a slightly higher BMI percentile than their younger peers (99.1 *versus *98.0, respectively; p = 0.03).

**Table 1:** Comparison of demographic and anthropometric data of clients referred for pediatric weight management living within or beyond the Edmonton Census Metropolitan Area (ECMA).

	Within ECMA (n = 455)	Beyond ECMA (n = 100)	p-value
**Sex (n; %)**	Boys (n = 232; 51%) Girls (n = 223; 49%)	Boys (n = 54; 54%) Girls (n = 46; 46%)	-
**Age (y)**	12.4 ± 2.6	12.4 ± 2.7	0.9
**Height (cm)**	155.2 ± 14.3	159.0 ± 15.2	0.02
**Weight (kg)**	78.0 ± 24.6	92.4 ± 33.2	<0.001
**BMI (kg/m**^**2**^**)**	31.6 ± 6.1	35.4 ± 8.6	<0.001
**BMI Percentile**	98.3 ± 1.8	98.9 ± 1.3	0.001
**BMI Z-Score**	2.24 ± 0.35	2.43 ± 0.36	<0.001

Data are presented as proportions and means ± standard deviation.

## Discussion and Conclusions

There are clear rural-urban inequities regarding health care access and treatment outcomes. We add to this body of research by examining proximity in relation to pediatric weight management. This elaboration is warranted for several reasons. First, it provides a basic understanding of the differences between distant and local clients referred for weight management. Second, the demand for pediatric weight management as defined by geographic scope is not well known. Finally, it draws attention to the need to define how health services delivery must be structured to best meet clients' needs. To optimize access to and management outcomes from specialized pediatric weight management services, a thorough characterization of these issues is essential. In our study, the majority of physician referrals were for obese or very obese clients. While most clients lived locally, approximately one in five lived beyond ECMA. This distinction proved important since distant clients were more overweight than their local counterparts, a finding that highlights the importance of developing adaptable models of care for managing pediatric obesity since a *one size fits all *approach will neither be appropriate nor feasible for families in different geographic locations.

Despite the recommendation that boys and girls should routinely have their BMI plotted on a growth chart [9,10], most overweight children are not being screened by physicians [11]. Since physicians are more likely to discuss weight management with their patients in cases of severe obesity [12], an outstanding need remains to raise clinicians' awareness regarding the value in and process of referring both overweight and obese clients for weight management care. Program leaders from specialized centres can also take a proactive approach by initiating opportunities for collaboration with colleagues, especially those practicing in the primary care setting. By identifying areas of strength (i.e., primary care may be more accessible for families) and limitation (i.e., physicians may have low levels of perceived competence in weight management care), program leaders can help to establish and enhance local referral and clinical support systems for weight management to better meet the needs of clinicians and clients, irrespective of geographic location.

On average, our distant clients weighed ~14 kg more and had a BMI approximately four units greater than their local peers, which suggests they may be at increased health risk. This is particularly relevant for distant youth since they tended to be more overweight than their younger peers. The etiology of these discrepancies is unknown; however, multiple mechanisms may be involved, including both social causation and social selection effects [13]. In addition, physicians' likelihood of referring clients for weight management may vary by proximity; for example, in relation to physicians within ECMA, clinicians working in rural or remote communities may be more inclined to encourage families to access local services or programs for weight management, especially when obesity is less severe. This dichotomy may help to explain why distant clients were heavier and more overweight than their local counterparts.

The number of referrals we received for clients living in distant communities demonstrated that weight management care must be flexible depending on clients' needs and capacity. To date, the evidence supporting the successful management of pediatric obesity has been derived primarily from group-based, lifestyle interventions [14], a means of health services delivery that is not feasible for most individual families residing in rural and remote communities. Distance can also represent a barrier when frequent in-person contact is required for intensive weight management interventions (i.e., bariatric surgery) that require extended support from specialized health care teams. Therefore, alternative forms of communication (i.e., videoconferencing) may be more practical for families and clinicians, regardless of treatment modality. Although these technologies hold promise [15-17], additional research is needed to evaluate the effectiveness of e-health interventions to improve process and clinical outcomes in pediatric weight management.

Despite the strength of having an inclusive dataset containing all referrals received since our program inception, analyses were limited by the information provided by referring physicians on the one-page referral form. Additional demographic and medical data from clients were not available, but these details can inform weight management intervention foci and centres' resource allocation. Given reports of high levels of intervention attrition [18,19], contextual details can help to guide the delivery of obesity-related health services. Research exploring families' decisions regarding the initiation of pediatric weight management care post-referral will offer valuable insight. This is especially relevant with respect to the local-distant dichotomy since proximity may be a surrogate measure of more salient factors (i.e., motivation to make lifestyle and behaviour changes) that influence weight management success.

## Competing interests

The authors declare that they have no competing interests.

## Authors' contributions

KAA conceived of the study, participated in its design and coordination, assisted with data analysis, and co-authored the first draft of the manuscript. DWJH helped conceive of the study and performed the spatial distribution/geographic data analysis. GDCB helped conceive of the study, participated in the study design and coordination, led the data analysis, and co-authored the first draft of the manuscript. All authors read, edited, and approved the final manuscript.

## Pre-publication history

The pre-publication history for this paper can be accessed here:

http://www.biomedcentral.com/1472-6963/10/302/prepub
